# Vulvovaginal Candidiasis in Pregnancy: Species Distribution and Antifungal Resistance Patterns to Commonly Used Topical Azoles at a Tertiary Care Center in Central India

**DOI:** 10.7759/cureus.114835

**Published:** 2026-08-20

**Authors:** Arati A Bhadade, Tadepalli Karuna, Bharti Singh, Anirban Ramani, Gajendra Singh, Madhu Panthi, Ankita Beniwal, Neha Upadhyay, Swathi S

**Affiliations:** 1 Microbiology, All India Institute of Medical Sciences, Bhopal, Bhopal, IND; 2 Obstetrics and Gynecology, All India Institute of Medical Sciences, Bhopal, Bhopal, IND

**Keywords:** candida albicans, clotrimazole, miconazole, minimum inhibitory concentration, non-albicans candida, pregnancy, vulvovaginal candidiasis

## Abstract

Introduction: Vulvovaginal candidiasis (VVC) is a common fungal infection during pregnancy. Topical azoles, including clotrimazole and miconazole, are recommended as first-line therapy; however, data on the distribution of *Candida* species and their in vitro susceptibility to these agents remain limited. This study aimed to determine the species distribution of *Candida* causing VVC in pregnant women and evaluate the in vitro activity of clotrimazole and miconazole. Materials and methods: This six-month observational cross-sectional study was conducted at a tertiary care center in Central India. High vaginal swabs were collected from 108 symptomatic pregnant women and examined by direct microscopy and culture. *Candida* isolates were identified using matrix-assisted laser desorption/ionization time-of-flight mass spectrometry. Antifungal susceptibility testing for clotrimazole and miconazole was performed using the Etest method. Minimum inhibitory concentration (MIC) values were analyzed descriptively because clinical breakpoints (CBPs) and epidemiological cutoff values (ECVs) for these topical azoles were unavailable. Descriptive statistics included frequencies, percentages, mean, median, mode, and MIC ranges. Results: *Candida* species were isolated from 45 of 108 pregnant women (41.67%). *Candida albicans* was the predominant isolate (36/45, 80.0%), followed by *Nakaseomyces glabrata* (4/45, 8.89%), *Candida tropicalis* (2/45, 4.44%), *Pichia kudriavzevii* (1/45, 2.22%), *Candida parapsilosis* (1/45, 2.22%), and *Candida lusitaniae* (1/45, 2.22%). Clotrimazole demonstrated lower MIC values than miconazole. Higher MIC values (>32 µg/mL), indicating reduced in vitro activity, were observed in nine (25.0%) *Candida albicans* isolates with miconazole, compared with one (2.78%) with clotrimazole.

Conclusions: *Candida albicans* remained the predominant cause of VVC among pregnant women. Clotrimazole demonstrated lower MIC values than miconazole in this study, indicating greater in vitro activity based on the observed MIC distribution. Continued surveillance of antifungal susceptibility patterns and the establishment of validated CBPs for topical azoles against *Candida* species are essential for improving understanding of emerging antifungal resistance and supporting evidence-based management of VVC.

## Introduction

Vulvovaginal candidiasis (VVC) is a significant global health concern that affects millions of women, causing considerable morbidity and negatively impacting quality of life. Approximately 70-75% of women experience VVC at least once in their lifetime [1], with symptoms such as abnormal vaginal discharge (“cottage cheese/white curd-like discharge”), pruritic vaginal discomfort, dyspareunia, and dysuria [2,3]. Pregnancy is a major risk factor for VVC, along with diabetes mellitus, contraceptive use, broad-spectrum antibiotic use, and HIV infection [4]. During pregnancy, the incidence of VVC is increased due to the weakened immune system and factors such as increased production of vaginal mucosal glycogen, increased estrogen levels, low vaginal pH, and decreased cell-mediated immunity [5,6]. VVC during pregnancy has been associated with complications, including preterm birth, chorioamnionitis with subsequent abortion, premature rupture of membranes, and superficial mucocutaneous infections, such as oral thrush and diaper dermatitis in the neonates [6,7].

*Candida albicans* remains the predominant pathogen (85-95%) causing VVC. Non-albicans *Candida* (NAC) species are increasingly reported, especially in recurrent or treatment-resistant cases [8]. Common NAC species associated with VVC include *Nakaseomyces glabrata*, *Pichia kudriavzevii*, *Candida tropicalis*, *Candida parapsilosis*, *Nakaseomyces nivariensis*, *Meyerozyma guilliermondii*, *Candida orthopsilosis*, and *Candida metapsilosis* [7,9]. To manage VVC, especially in cases resistant to treatment, antifungal susceptibility testing (AFST) is essential [10].

Topical azoles such as clotrimazole and miconazole are considered standard therapeutic options for pregnant women. According to the Centers for Disease Control and Prevention, topical azole therapies are recommended as first-line treatment for VVC during pregnancy, with a treatment duration of at least seven days to ensure optimal efficacy. Systemic fluconazole, even a single 150 mg dose, has been associated with spontaneous abortion and congenital anomalies, as indicated by epidemiologic studies, and is contraindicated during pregnancy. In contrast, topical azoles, when used at conventional doses, have not been linked to an increased risk of major congenital malformations. Hence, they are considered the safest and most effective treatment option during pregnancy [11-14]. Although oral fluconazole is considered the drug of choice for VVC, it is generally not used during pregnancy. Therefore, most clinicians rely on local antifungal therapy, such as clotrimazole or miconazole preparations. The antifungal susceptibility patterns for these drugs are less well studied because the clinical breakpoints (CBPs) or epidemiological cutoff values (ECVs) are not available. After an extensive literature search, we found very limited data on antifungal resistance patterns for clotrimazole and miconazole in *Candida* species isolated from VVC [9,15].

By understanding the distribution and antifungal susceptibility of *Candida* species, clinicians can provide pathogen-targeted treatment and improve patient outcomes. Hence, this study aimed to determine the species distribution and in vitro activity of commonly used topical azoles for VVC during pregnancy at a tertiary care center.

## Materials and methods

Study design and setting

A six-month observational cross-sectional study was conducted in the Departments of Microbiology and Obstetrics and Gynecology (OBG) at a tertiary care center in central India from August 2025 to January 2026, with approval from the Institutional Human Ethics Committee - Student Research of All India Institute of Medical Sciences, Bhopal (approval number: Ihecsr/aiimsbpl/July25/08).

Study population and sample size

The study included all symptomatic pregnant women of reproductive age (18-45 years) with clinically suspected VVC who presented to the OBG outpatient department (OPD) during the study period, during any trimester of pregnancy. Collected high vaginal swab (HVS) samples were sent for fungal culture testing in the microbiology laboratory. The study excluded duplicate isolates from the same patients and patients who declined to participate or provide informed consent.

Sample collection procedure

**Figure 1:**
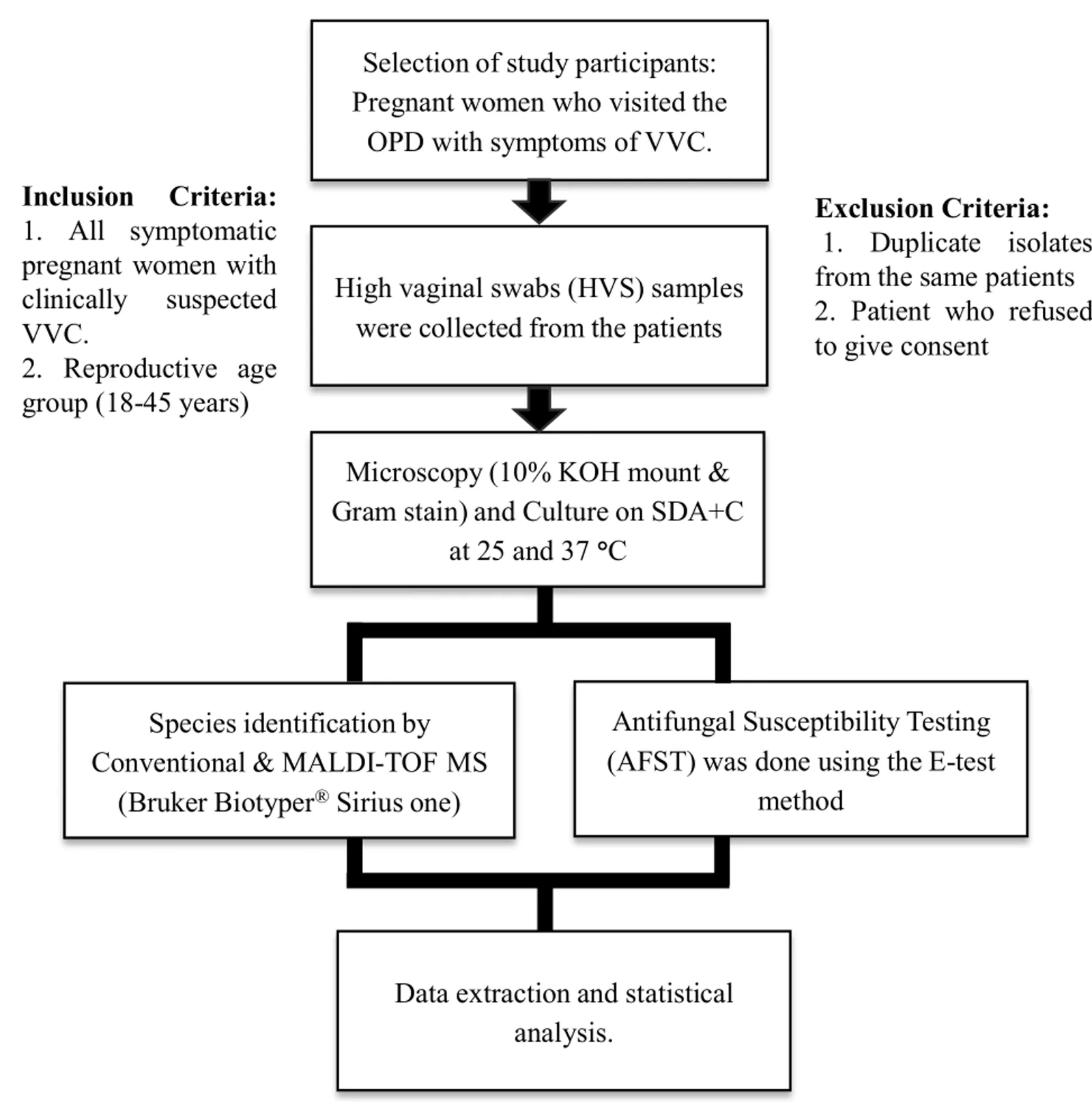
Workflow of the study OPD: outpatient department, VVC: vulvovaginal candidiasis, HVS: high vaginal swab, KOH: potassium hydroxide, SDA: Sabouraud Dextrose Agar, C: chloramphenicol, MALDI-TOF MS: matrix-assisted laser desorption/ionization time-of-flight mass spectrometry, AFST: antifungal susceptibility testing

Study participants were enrolled in the OPD after fulfilling the inclusion and exclusion criteria. Samples were collected after patients provided written informed consent. The patients were placed in the lithotomy position, and 2% lignocaine was applied to the vulval introitus and the speculum to relieve discomfort. A sterile Cusco speculum was gently inserted under aseptic conditions. The vaginal walls were inspected for discharge and inflammation using a light source. Using a sterile polyester-tipped (Dacron) swab stick, samples were collected from the posterior vaginal fornix. Two HVS samples were obtained from each participant. The speculum was removed gently. Specimens were properly labeled and transported to the microbiology laboratory within two hours of collection. The study workflow is shown in Figure 1.

Laboratory process

**Figure 2:**
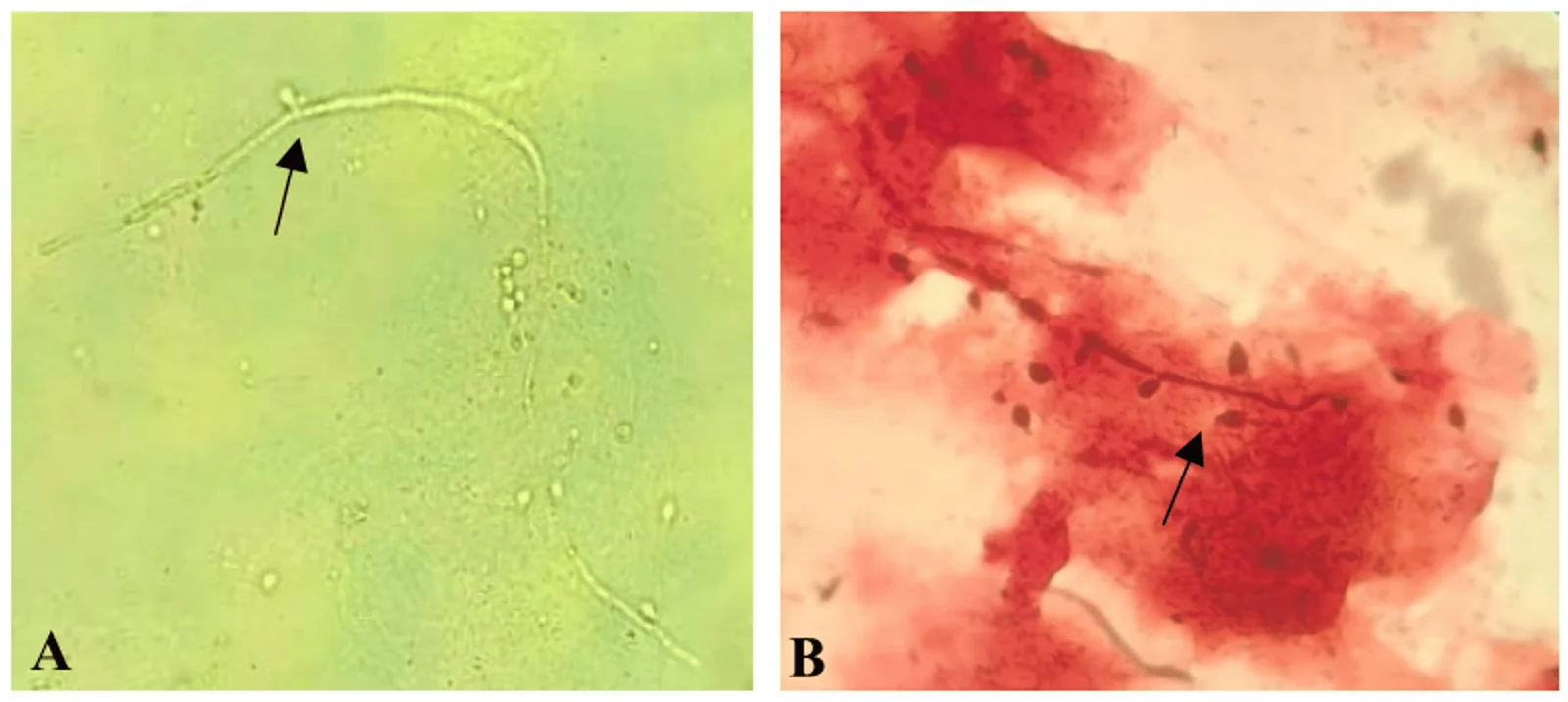
Microscopic examination Microscopic examination of an HVS: (A) 10% KOH mount; the arrow shows BYC with pseudohyphae, and (B) Gram stain; the arrow shows Gram-positive BYC with pseudohyphae. KOH: potassium hydroxide, BYC: budding yeast cell, HVS: high vaginal swab

Two HVS samples were collected from each patient: one for microscopy and one for culture and species identification. For direct microscopic examination, a 10% potassium hydroxide (KOH) wet mount and Gram staining were performed using two prepared smears from the HVS sample. The slides were examined under a microscope to detect the fungal elements, including budding yeast cells (BYC) and pseudohyphae. The findings of microscopic examination are shown in Figure 2.

HVS samples were inoculated onto two Sabouraud Dextrose Agar (SDA) plates with chloramphenicol (Himedia Laboratories). Inoculated plates were incubated for 24-48 hours at both 25ºC and 37ºC to assess fungal growth. Plates were examined after incubation. Creamy white, smooth, and pasty colonies were seen on SDA with chloramphenicol. All *Candida* isolates were identified by matrix-assisted laser desorption/ionization time-of-flight mass spectrometry (MALDI-TOF MS; Bruker Daltonics, Bremen, Germany). A MALDI score of ≥2.0 was considered high-confidence species-level identification; a score of 1.70-1.99 was interpreted as genus-level identification, and scores < 1.70 were interpreted as no reliable identification.

For all identified *Candida* isolates, AFST was performed using the gradient diffusion method (Etest) (Ezy MICTM Strips, HiMedia Laboratories Pvt. Ltd., Thane, India) for topical azoles, including miconazole and clotrimazole, each with a concentration range of 0.002-32 µg/mL. AFST was performed on Mueller-Hinton agar with 2% glucose and 0.5 µg/mL methylene blue according to the manufacturer’s instructions. Fresh, pure *Candida* cultures were used to prepare the inoculum, and isolated colonies were suspended in sterile saline and adjusted to a 0.5 McFarland standard using a spectrophotometer. *Candida parapsilosis* ATCC 22019 was used as the quality control for the Etest. The MIC values were reported at the point where the edge of the inhibition ellipse intersects the scale on the strip. Results were not interpreted because CBPs and ECVs are unavailable for clotrimazole and miconazole against *Candida* species. MIC data were analyzed descriptively and were not categorized as susceptible, intermediate, resistant, wild-type, or non-wild-type.

Data analysis

A standardized data collection form was created for all samples, and the recorded variables were entered into an Excel spreadsheet (Microsoft Corp., Redmond, WA, USA) for analysis. Categorical variables, including age group, trimester of pregnancy, clinical symptoms, and *Candida* species distribution, were expressed as frequencies and percentages. Continuous variables, including minimum inhibitory concentration (MIC) values for clotrimazole and miconazole, were summarized using the median, geometric mean, and mode MIC values because the MIC distributions were not normally distributed. No inferential statistical comparison of MIC values between antifungal agents was performed because the absence of validated interpretive criteria precludes clinically meaningful statistical comparisons.

## Results

Over the course of six months, 108 pregnant women with symptoms and clinical signs of VVC were enrolled in the study. All samples were processed according to the established laboratory protocol. Out of the 108 HVS samples analyzed, 45 (41.67%) tested positive for *Candida* species.

The largest proportion of culture-positive cases occurred in the 26-35-year age group (29, 64.4%), followed by the 18-25-year age group (12, 26.7%), and the 36-45-year age group (4, 8.9%). The distribution of species showed a similar pattern, with NAC and *Candida albicans* both being more frequently identified in the 26-35-year age group. Among 45 culture-positive cases, the third trimester had the highest percentage (27, 60%), followed by the second trimester (14, 31.1%) and the first trimester (4, 8.9%). Both NAC species and *Candida albicans* were most frequently identified in the third trimester.

All culture-positive patients presented with abnormal vaginal discharge, the most common symptom of VVC. The other symptoms, including itching (39, 86.66%), irritation (24, 53.33%), burning (23, 51.11%), dysuria (16, 35.56%), redness (11, 24.44%), swelling (7, 15.56%), and dyspareunia (4, 8.89%), were recorded.

**Figure 3:**
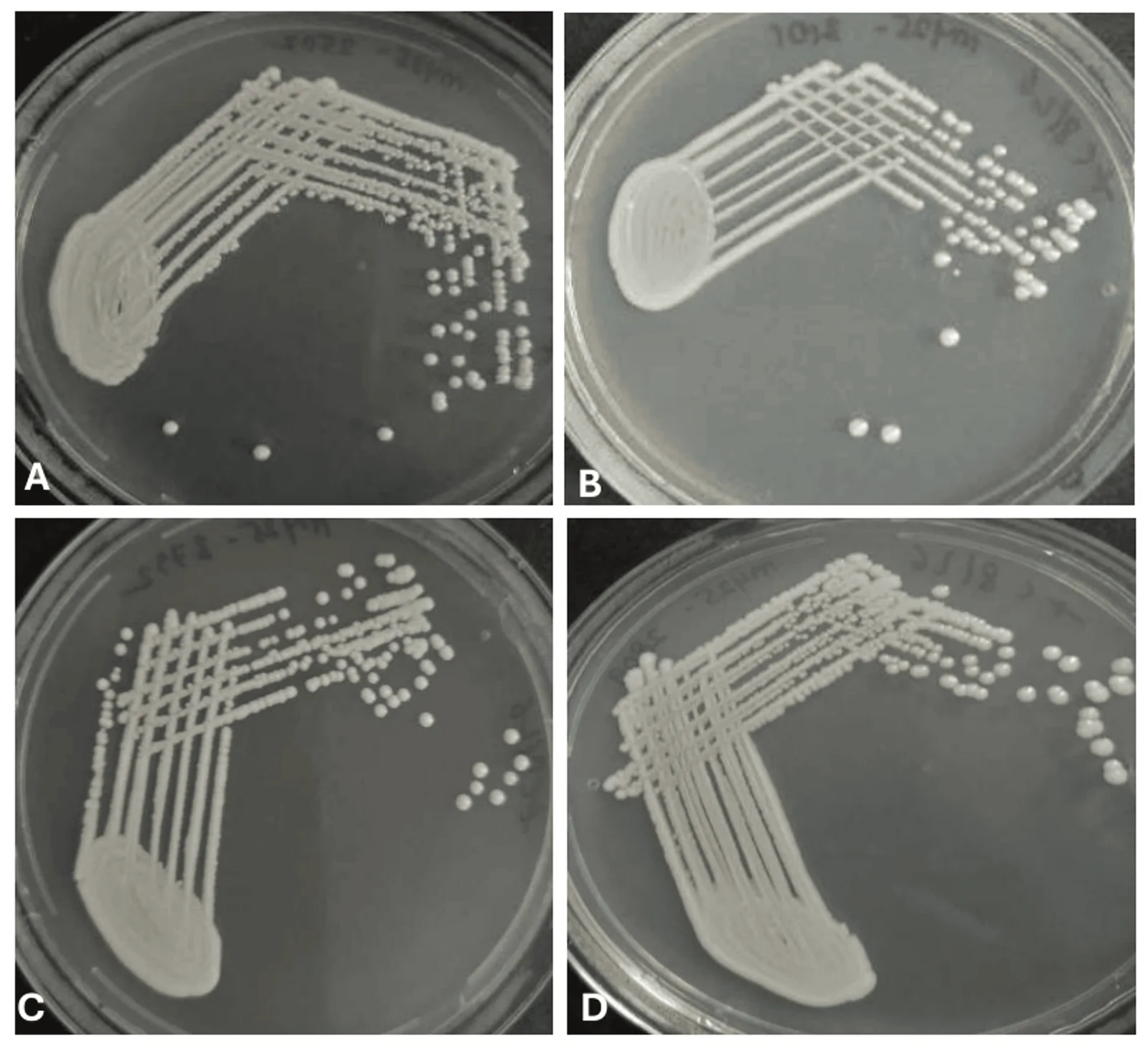
Culture on Sabouraud Dextrose Agar with chloramphenicol showing growth of the Candida isolates: (A) Candida albicans, (B) Nakaseomyces glabrata, (C) Candida lusitaniae, and (D) Candida tropicalis

The growth characteristics of the isolated *Candida* species are shown in Figure 3. As shown in Table 1, the most commonly isolated species was *Candida albicans* (36, 80%). Among the NAC species, the most frequently isolated species were *Nakaseomyces glabrata* (4, 8.89%), followed by *Candida tropicalis* (2, 4.44%), *Pichia kudriavzevii* (1, 2.22%), *Candida parapsilosis* (1, 2.22%), and *Candida lusitaniae* (1, 2.22%).

**Table 1:** Distribution of Candida species isolated from symptomatic cases of VVC (n = 45) VVC: vulvovaginal candidiasis

*Candida s*pecies	Frequency (n = 45) (%)
Candida albicans	36 (80%)
Nakaseomyces glabrata	4 (8.89%)
Candida tropicalis	2 (4.44%)
Pichia kudriavzevii	1 (2.22%)
Candida parapsilosis	1 (2.22%)
Candida lusitaniae	1 (2.22%)

**Figure 4:**
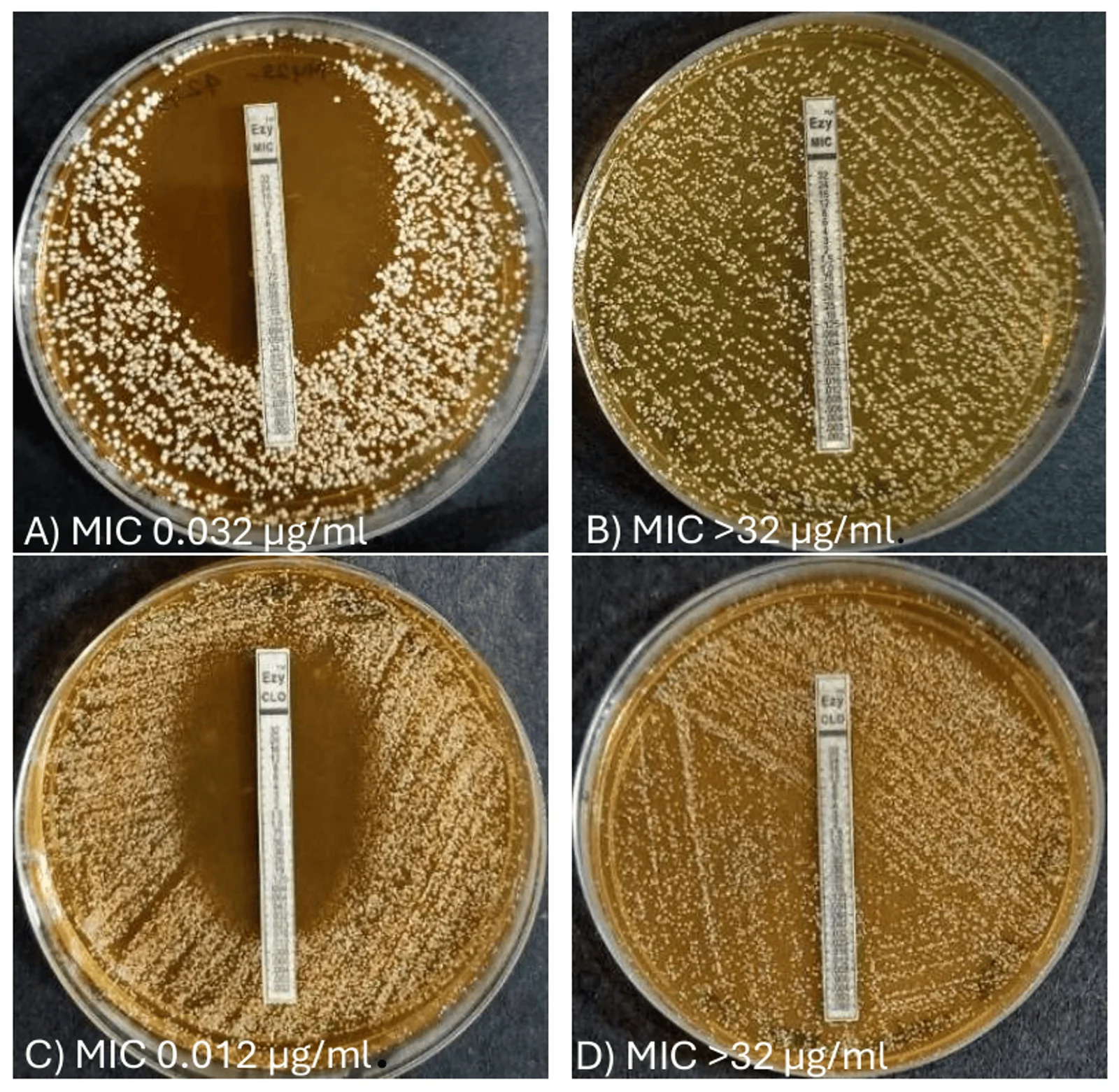
AFST by Etest (A) Miconazole showing a wide zone of inhibition with an MIC of 0.032 µg/mL; (B) miconazole showing no zone of inhibition, with an MIC of >32 µg/mL; (C) clotrimazole showing a wide zone of inhibition with an MIC of 0.012 µg/mL; and (D) clotrimazole showing no zone of inhibition, with an MIC of >32 µg/mL. MIC: minimum inhibitory concentration, AFST: antifungal susceptibility testing

Figure 4 illustrates the inhibition of miconazole and clotrimazole against *Candida* isolates by the Etest method. The MICs of miconazole and clotrimazole against *Candida* isolates ranged from 0.016 to >32 µg/mL and 0.006 to >32 µg/mL, respectively. The median MICs were 0.125 µg/mL and 0.016 µg/mL, while the geometric mean MICs were 0.39 µg/mL and 0.042 µg/mL, respectively. The most frequently observed MICs for miconazole were 0.064 µg/mL (frequency: 10), >32 µg/mL (frequency: 9), and 0.125 µg/mL (frequency: 8). For clotrimazole, the most frequent MICs were 0.016 µg/mL (frequency: 13) and 0.012 µg/mL (frequency: 9), as shown in Table 2.

**Table 2:** Descriptive statistics for MIC values of topical azoles against Candida isolates determined by the Etest MIC: minimum inhibitory concentration

	Miconazole	Clotrimazole
Manufacturer specified Etest MIC range	0.002->32 µg/mL	0.002->32 µg/mL
Observed MIC range among study isolates	0.016->32 µg/mL	0.006->32 µg/mL
Geometric mean MIC	0.39 µg/mL	0.042 µg/mL
Median MIC	0.125 µg/mL	0.016 µg/mL
Mode MIC	0.064 (10), >32 (9), 0.125 (8) µg/mL	0.016 (13), 0.012 (9) µg/mL

All 45 *Candida* isolates underwent AFST by the Etest method. Based on Etest MIC values, *Candida albicans* and NAC isolates were analyzed descriptively. MICs >32 µg/mL were considered to reflect reduced in vitro activity for descriptive purposes, whereas lower MICs suggest greater in vitro activity. As shown in Table 3, of the 36 *Candida albicans* isolates tested, nine (25.0%) showed higher MICs against miconazole and one (2.78%) against clotrimazole, indicating reduced in vitro activity. Similarly, both topical azoles showed greater in vitro activity against all nine NAC isolates. Based on these results, among the 45 isolates tested, miconazole showed markedly reduced in vitro activity compared with clotrimazole. The MIC results are presented descriptively, without being divided or interpreted into susceptible or resistant groups, because CBP and ECVs for miconazole and clotrimazole against *Candida* species are currently unavailable. Baseline information on the in vitro activity of miconazole and clotrimazole against *Candida* isolates in this study is provided by the distribution of MIC values. The MIC data for all isolates against these two drugs are provided in Table 3, which may serve as a useful reference for future research and for the establishment of CBPs by expert scientific committees.

**Table 3:** MIC distribution and in vitro antifungal activity for miconazole and clotrimazole against Candida isolates (n = 45) MIC: minimum inhibitory concentration, NAC: non-albicans *Candida*

*Candida *species	MIC values	Miconazole MIC (µg/ml) values (n = 45) (%)	Clotrimazole MIC (µg/ml) values (n = 45) (%)	Interpretation
Candida albicans	>32	9 (25.0)	1 (2.78)	Higher MIC, reduced in vitro activity
	1.5	1 (2.78)	-	Greater in vitro activity
	0.75	-	1 (2.78)	
	0.50	1 (2.78)	2 (5.55)	
	0.25	1 (2.78)	4 (11.11)	
	0.19	4 (11.11)	-	
	0.125	6 (16.66)	-	
	0.094	1 (2.78)	2 (5.55)	
	0.064	9 (25.0)	2 (5.55)	
	0.047	2 (5.55)	1 (2.78)	
	0.032	1 (2.78)	1 (2.78)	
	0.023	-	1 (2.78)	
	0.016	1 (2.78)	11 (30.56)	
	0.012	-	9 (25.0)	
	0.006	-	1 (2.78)	
NAC				
Nakaseomyces glabrata	0.75	1 (11.11)	-	Greater in vitro activity
	0.25	1 (11.11)	1 (11.11)	
	0.125	1 (11.11)	1 (11.11)	
	0.094	-	1 (11.11)	
	0.064	1 (11.11)	-	
	0.032	-	1 (11.11)	
Candida tropicalis	0.38	1 (11.11)	-	
	0.19	1 (11.11)	-	
	0.047	-	1 (11.11)	
	0.032	-	1 (11.11)	
Pichia kudriavzevii	0.50	1 (11.11)	-	
	0.016	-	1 (11.11)	
Candida parapsilosis	1	1 (11.11)	-	
	0.023	-	1 (11.11)	
Candida lusitaniae	0.125	1 (11.11)	-	
	0.016	-	1 (11.11)	

## Discussion

VVC is a common fungal infection of the female genital tract caused by *Candida* species. Pregnancy is considered a significant predisposing factor due to hormonal changes. During pregnancy, elevated hormonal levels stimulate the vaginal epithelium to accumulate glycogen. This increased glycogen content can act as a readily available carbon source, creating a favorable environment for the growth and multiplication of *Candida* species [16].

A culture positivity rate of 41.67% was observed among symptomatic pregnant women in this study. Recent research also shows significant variation in the prevalence of VVC among pregnant women. In contrast to our study, studies by Siddiqui et al. and Roy et al. reported a lower prevalence of 32.7% and 35% among women presenting with vaginitis at a tertiary care center in central India [17,18]. These findings suggest that the burden of VVC during pregnancy remains considerable and may vary by geographic region, study populations, and diagnostic methods [19]. The overall positivity rate of 41.67% observed in this study may be due to several factors. First, the study group included only symptomatic pregnant women, which increased the likelihood of detecting an active infection. Additionally, immune changes during pregnancy may promote *Candida* overgrowth and persistence in the vaginal area.

In the present study, the highest prevalence (64.4%) of VVC was observed in the 26-35-year age group. A similar finding was reported by Christopher et al., who found the highest prevalence of 56.7% among pregnant women [20]. This increased prevalence may be related to hormonal changes, elevated estrogen levels, and increased glycogen deposition during pregnancy, all of which favor *Candida* growth.

Our study indicates that a higher proportion of VVC cases (60%) occurs among women in the third trimester of pregnancy. A similar trend was reported by Moursi et al., who documented that 43.7% of VVC cases occurred during the third trimester [21]. These findings suggest that VVC may be more frequently detected in later pregnancy.

Clinically, all culture-positive individuals in this study had abnormal vaginal discharge (curdy white discharge), along with the symptoms of itching and irritation. A study by Mariyah et al. found that pruritus and curdy white discharge were the most common symptoms [22].

Our study demonstrated that *Candida albicans* accounted for 80% of isolates, confirming it as the predominant etiological agent of VVC. A study by Gedefie et al. also reported similar results, with a 63.3% isolation rate of *Candida albicans* [23]. However, the presence of 20% NAC species in this study also highlights the increasing contribution of NAC species to VVC. A similar increase in the prevalence of NAC species has been documented in recent studies [7,24]. The predominance of *Candida albicans* may be related to its greater virulence and ability to colonize vaginal epithelium. In contrast, NAC species are increasingly recognized due to their emerging clinical significance and reduced susceptibility to azole antifungals.

Topical azoles, such as clotrimazole and miconazole, remain important first-line treatments within the azole class for VVC in pregnant women because of their proven efficacy, low systemic absorption, and favorable safety profile. In our study, the median MIC and mode MIC for clotrimazole were both 0.016 µg/mL. The median and mode MICs for miconazole were 0.125 µg/mL and 0.064 µg/mL, respectively. The geometric mean MICs were 0.39 µg/mL for miconazole and 0.042 µg/mL for clotrimazole. These values were not interpreted as susceptible or resistant according to CLSI or EUCAST guidelines. Still, they were presented descriptively because CBPs and ECVs are currently unavailable for these drugs in any standard guidelines. In our study, the results suggest that among 36 *Candida albicans* isolates, reduced in vitro activity was observed for miconazole and clotrimazole in 25.0% and 2.78% of isolates, respectively, indicating that miconazole exhibited markedly reduced activity compared to clotrimazole. A study by Phung et al. found high susceptibility to clotrimazole (108 cases) and miconazole (41 cases) among *Candida albicans* isolates, with five cases resistant to miconazole and one case resistant to clotrimazole [25]. Similarly, a 2025 study by Bowen et al. found that *Candida albicans* showed 47.1% reduced susceptibility to miconazole and 41.4% reduced susceptibility to clotrimazole [26], highlighting population differences and the need for ongoing monitoring and susceptibility testing to ensure safe and effective therapy. Resistance to clotrimazole and miconazole in *Candida* species can be associated with mutations in the ERG11 gene encoding the target enzyme lanosterol 14-α-demethylase, overexpression of efflux transporters, alterations in ergosterol biosynthesis pathways, biofilm formation, and chromosomal adaptations that increase the expression of resistance-related genes [27,28].

Topical azole antifungals, particularly clotrimazole and miconazole, constitute the mainstay of empirical treatment for VVC during pregnancy, as oral fluconazole is generally avoided because of potential fetal risks. In the present study, reduced in vitro susceptibility to miconazole was observed among *Candida* isolates, whereas clotrimazole demonstrated comparatively better in vitro activity. This study has several strengths, including its clinically relevant focus on VVC in pregnancy, prospective enrollment of symptomatic pregnant women over a defined study period, species-level identification of *Candida* isolates using MALDI-TOF MS, and assessment of the in vitro activity of commonly used topical azoles based on MIC values.

This study has certain limitations. The study was conducted at a single tertiary care center over a relatively short study period. It included a limited number of symptomatic pregnant women, which may limit the generalizability of the findings. No formal a priori sample-size calculation was performed, as all eligible participants presenting during the predefined study period were included. The analysis was primarily descriptive, and no inferential statistical analyses were performed. Patient follow-up was not performed; therefore, treatment response, clinical outcomes, and recurrence could not be assessed, and the relationship between MIC values and treatment outcomes could not be established. Additionally, because validated CBPs and ECVs are unavailable for clotrimazole and miconazole against *Candida* species, MIC values were reported/analyzed descriptively and were not categorized as susceptible or resistant.

## Conclusions

This study offers valuable insights into the current epidemiology and in vitro antifungal activity patterns of VVC in pregnant women. A high rate of *Candida* culture positivity was observed among symptomatic pregnant women, with *Candida albicans* remaining the predominant etiological agent. However, a substantial proportion of infections were caused by NAC species, particularly *Nakaseomyces glabrata*. Among the commonly used topical antifungal agents, clotrimazole demonstrated lower MIC values against *Candida albicans* isolates than miconazole, indicating greater in vitro activity in this study; however, clinical efficacy cannot be inferred from these MIC findings alone. These findings highlight the importance of continuous local surveillance of antifungal susceptibility patterns and support the use of AFST, particularly in patients with persistent or recurrent infections or those failing empirical therapy. Furthermore, the lack of established CBPs for widely used topical antifungal agents, such as clotrimazole and miconazole, limits the clinical interpretation of susceptibility data. Development of standardized CBPs for these agents is warranted to improve the understanding of emerging antifungal resistance and to guide evidence-based management of VVC.
